# Financial strain contributes to daily depressive and anxiety symptoms in Hispanic and Latino family caregivers of people living with dementia: A daily diary study

**DOI:** 10.1002/bsa3.70095

**Published:** 2026-07-13

**Authors:** Kylie Meyer, Natashia Bibriescas, Abigail Poe, Megan Thomas Hebdon, Frank Puga

**Affiliations:** 1 Frances Payne Bolton School of Nursing, Case Western Reserve University, Cleveland, Ohio, USA; 2 School of Nursing, University of Alabama at Birmingham, Birmingham, Alabama, USA; 3 College of Nursing, University of Utah, Salt Lake City, Utah, USA; 4 Department of Family Medicine, Keck School of Medicine of USC, Los Angeles, California, USA

**Keywords:** caregiving, dementia, financial strain, Hispanic/Latino, psychological distress

## Abstract

**BACKGROUND:**

Family caregivers of people living with dementia (PLWD) face significant financial strain, which may adversely affect their mental health. While prior research links financial strain to depression and anxiety, few studies have explored its day-to-day impact, particularly among Hispanic and Latino (H&L) caregivers who experience high levels of financial strain.

**METHODS:**

This study used data from the *Nuestros Días* (Our Days) Study to examine daily mental health symptoms among H&L caregivers to PLWD (*N* = 186). Financial strain was assessed in a baseline survey, followed by 21 consecutive daily surveys measuring daily depressive and anxiety symptoms.

**RESULTS:**

Multi-level modeling revealed that higher financial strain was associated with daily depressive (odds ratio [OR] = 1.75, 95% CI: [1.07, 2.86]) and anxiety symptoms (OR = 1.75, CI: [1.09, 2.81).

**DISCUSSION:**

Interventions to alleviate mental health symptoms in this population should consider addressing financial strain as a unique source of caregiver distress.

## 1 | INTRODUCTION

There are approximately 12 million family caregivers to people living with Alzheimer’s disease and related dementias (ADRD) in the United States.^1^ The informal care provided to people living with dementia (PLWD) by family is estimated to be worth $233 billion.^2^ As the prevalence of ADRD continues to rise, the financial value and replacement costs are expected to increase substantially. Specifically, the annual replacement cost of care provided by family caregivers overall is estimated to increase from $182 billion to as much as $571 billion by 2060. Further, the proportion of these replacement costs borne by family caregivers of PLWD is expected to increase from 44% to 53% from 2011 to 2060.^3^

Despite the high value of care they provide, family caregivers of PLWD experience a very high financial burden, with over 40% reporting financial difficulty.^4,5^ This burden reflects both the direct costs of caregiving and its impacts on employment. Family caregivers of PLWD spend an average of $8978 annually on out-of-pocket caregiving expenses (e.g., home modifications, over-the-counter medications), compared to $7242 among caregivers of individuals living with any condition – a 24% difference.^6^ Notably, caregiving costs extend beyond medical treatment costs and include day-to-day living costs not covered by insurance.^7,8^ In addition, approximately two-thirds of caregivers of PLWD report that caregiving has negatively affected their work, including coming in late, leaving early, or taking time off. ^9^

Importantly, the financial challenges of caregiving are not equally distributed across populations. Hispanic and Latino (H&L) caregivers of individuals with any chronic or disabling condition spend an estimated 47% of their annual household income on out-of-pocket care expenses, compared to 26% among caregivers of any ethnicity.^6^ H&L families also report more negative financial impacts from caregiving, such as increased debt, reduced savings, and higher financial strain. For example, 31% of H&L caregivers report accruing debt due to caregiving, compared to 23% of caregivers overall.^10^ H&L caregivers also consistently identify financial strain as one of the greatest sources of stress, often describing a sense of obligation to spend money on care regardless of their financial capacity.^8,11^ Moreover, Hispanics and Latinos are at a greater risk of experiencing ADRD relative to non-Hispanic Whites, which may increase the financial impacts on this population. For example, ADRD prevalence at ages 80 and older is 40.2% among Hispanics and Latinos, compared to 33.6% among non-Hispanic Whites.^12^

Despite this disproportionate burden, few studies have examined how financial strain affects the mental health of H&L ADRD family caregivers. This study uses data from an ongoing daily diary survey study to examine associations between financial strain and daily depressive and anxiety symptoms in a national sample of H&L ADRD family caregivers. By examining how financial strain associates with day-to-day psychological distress (i.e., depressive and anxiety symptoms), this study aims to elucidate how financial hardship shapes the daily experiences of H&L ADRD family caregivers.

### 1.1 | Background: the role of subjective financial strain on caregiver outcomes

#### 1.1.1 | Subjective financial strain and the stress process model

Subjective financial strain refers to an individual’s perception of their financial resources, as opposed to material financial strain, which includes objective costs and effects on income and assets.^13^ Prior research among family caregivers suggests subjective income inadequacy, a type of subjective financial strain, is a stronger predictor of depression than actual household income.^4^ Subjective financial strain encompasses not only current perception of one’s financial well-being but also anticipated caregiving-related costs and worry about one’s financial future.^7,8,14^ Consistent with this literature, the term “financial strain” is used throughout this manuscript to refer specifically to “subjective financial strain.”

The Stress Process Model (SPM) has been widely applied to conceptualize the pathways through which caregiving stressors influence caregiver health outcomes, including physical and mental health.^15–17^ Within the SPM, primary stressors are those that result directly from caregiving (e.g., managing behavioral and psychological symptoms, assisting with activities of daily living and instrumental activities of daily living), while secondary stressors are indirectly related to care-giving (e.g., family conflict, competing job demands).^18^ When applied to financial contexts, objective caregiving costs may function as a primary stressor, while worry about paying bills may operate as a secondary stressor. As such, subjective financial strain is distinct but related to objective financial stressors.

The broader context in which caregiving occurs can further contribute to differential exposure to and impacts of stressors within the SPM framework. Qualitative studies suggest that H&L families often experience heightened concern about the financial consequences of family caregiving, including expectations to cover care-related expenses or to provide direct care when doing so compromises employment or financial stability.^7,8,11^ These patterns may reflect cultural values that emphasize familial responsibility and prioritize caring for older adults, even at substantial personal cost.^19,20^ When considered alongside disproportionately high objective financial stressors, H&L caregivers may be uniquely at risk of negative mental health outcomes related to financial strain.

#### 1.1.2 | Prior research on how financial strain impacts caregiver mental health

Relatively few studies directly examined associations between financial strain and depressive or anxiety symptoms among ADRD family caregivers. Two studies, drawing on data initially collected for the Resources for Enhancing Alzheimer’s Caregiver Health (REACH) study, examined this relationship directly. Sun et al. conducted a hierarchical regression analysis using a single-item measure of income adequacy and found that perceived income inadequacy was significantly associated with both caregiver anxiety and depression, whereas total household income was not.^4^ Nam extended these findings and found that financial difficulty predicted depressive symptoms.^21^ These results are consistent with longitudinal research among H&L older adults, though not necessarily caregivers, demonstrating that high financial strain predicts depressive symptoms.^22^

Longitudinal studies further support financial strain as a predictor of caregiver psychological distress. In a panel survey conducted every 4 months for 36 months with caregivers of PLWD in Singapore, Poco et al. found that greater financial difficulty was associated with higher caregiver psychological distress, poorer self-assessed health among caregivers, and poorer perceptions of quality of life among the PLWD.^5^ Using data from the Daily Stress and Health Study, Liu et al. reported that baseline financial strain predicted greater role overload and increasing perceived role captivity over time.^17^ In a daily survey study examining medical costs of care on a weekly basis, Dawson et al. found that paying for in-home respite care was associated with greater caregiver reactivity to behavioral and psychological symptoms of dementia (BPSDs) and higher caregiver burden at baseline.^23^ Given the disproportionate ADRD and caregiving burden among the H&L populations, these family caregivers may be at high risk of experiencing psychological distress related to financial strain.

### 1.2 | Limitations of prior research

To date, few studies have examined how financial strain affects daily depressive and anxiety symptoms among ADRD caregivers, despite heightened risk relative to other types of caregivers.^24^ A limitation of prior studies on caregiver mental health is their reliance on traditional, longer recall surveys that ask about symptom experience in the past month or several months, while mental health symptoms are known to fluctuate day to day.^25,26^ Daily measures are more ecologically valid and less susceptible to recall bias than traditional survey approaches.^27^ There are also important implications for practice: If financial strain affects caregivers’ daily mental health and well-being, mitigating it may improve family caregivers’ day-to-day quality of life. Finally, most existing studies examined experiences of caregiver financial strain in aggregate across populations, thereby masking how financial strain may worsen mental health in at-risk populations. This is a significant issue given that H&L ADRD caregivers experience higher levels of financial strain than non-Hispanic caregiving peers.

### 1.3 | Purpose of this study

The purpose of this study was to examine how perceived financial strain influences daily depressive and anxiety symptoms among H&L family caregivers of PLWD. The study focused specifically on H&L ADRD family caregivers in the United States, a population that is uniquely at risk of experiencing high financial strain and represents one of the fastest-growing populations impacted by dementia.^28^ We hypothesized that ADRD family caregivers who reported high levels of subjective financial strain at baseline would report more days with depressive and anxious symptoms compared to caregivers reporting lower financial strain.

## 2 | METHODS

### 2.1 | Procedure

This study was a cross-sectional analysis of data from the first wave of the *Nuestros Días* (Our Days) Study, an ongoing, national, intensive longitudinal study examining the daily mental health experiences of H&L ADRD family caregivers. Detailed information about the study design and procedures has been published elsewhere.^29^ Briefly, participants were recruited nationally from community settings, with the majority residing in Texas, Florida, and Puerto Rico. Data collection began in March 2023 and is ongoing. Participants completed surveys assessing contextual, individual-level, and cultural factors across three waves (enrollment, 6 months, and 12 months). Following each survey, participants completed 21 days of daily diary surveys capturing day-to-day caregiving experiences and well-being. Daily diary surveys were delivered each evening, and participants were instructed to complete them between 7:00 and 11:00 p.m. in their local time zone to reduce recall bias.

The analysis conducted includes data from the first 201 participants who completed the first wave of data collection (i.e., baseline survey and corresponding 21-day daily diary survey period following enrollment). Data for this analysis were collected between March 2023 and July 2025. All surveys were completed online using secure REDCap links sent to participant-provided email addresses.^30^ Participants could complete surveys on a computer, tablet, or smartphone. All participants received a study information sheet prior to participating in data collection. A signed consent form was waived due to the low-risk nature of the study. All procedures were approved by the University of Alabama at Birmingham Institutional Review Board (IRB-300008710).

### 2.2 | Participants

A convenience sample of H&L ADRD family caregivers was recruited nationally through social media platforms, printed advertisements, and community outreach, with materials available in English and Spanish. Eligibility criteria included age ≥18 years, self-identified as the primary caregiver of a family member living with ADRD, providing ≥4 h of unpaid care per week, and reliable Internet access. Caregivers were excluded if institutionalization of the care recipient was planned within 6 months or if the caregiver was terminally ill.

### 2.3 | Measures

For the present analysis, we used measures from the baseline survey and post-baseline daily diary surveys. All measures were available in English and Spanish, and participants completed surveys in their preferred language.

#### Sociodemographic characteristics.

At baseline, caregivers reported their age, sex, household income, and employment status. Caregivers also reported PLWD characteristics, including age, sex, insurance, and comorbid conditions (e.g., diabetes). Dementia and mild cognitive impairment status of the PWLD were assessed using the AD8.^31^

#### Financial strain.

Financial strain was measured at baseline using the financial subscale of the Caregiver Reaction Assessment.^32^ This measure characterizes financial strain as a distinct subcomponent of overall caregiver burden, which was identified using exploratory and confirmatory factor analyses. Inter-scale correlations demonstrate subscales of the Caregiver Reaction Assessment to be distinct from each other, as all correlations are under *r* = 0.40.^32^ Participants were asked to consider how much ofan impact caregiving had on their finances in the past 6 months. Items included: “Financial resources are adequate” (reverse coded), “It is difficult to pay for my relative,” and “Caring for my relative puts financial strain on me.” Items were rated on a 5-point Likert scale ranging from 1 (“strongly disagree”) to 5 (“strongly agree”). A total score was obtained by summing the three items, resulting in possible scores ranging from 3 to 15, with higher scores reflecting greater financial strain (Cronbach’s *α* = 0.63).

#### Primary caregiving stressors.

Primary caregiving stressors were operationalized as distress associated with BPSDs. Each evening, participants were asked the following question: “From 7:00 p.m. yesterday to 7:00 p.m. today, how BOTHERSOME did you find the following behaviors exhibited by your relative with memory problems or dementia?” The measure included 20 items covering domains such as restlessness, mood changes, memory-related symptoms, resistance to care, hallucinations and delusions, verbal and physical aggression, disinhibition, repetitive behaviors, safety concerns, and other bothersome behaviors. Items were rated on a 6-point Likert scale (0 = not at all bothersome to 5 = extremely bothersome), with higher scores indicating greater caregiving-related stress. If a BPSD did not occur on a given day, caregivers could indicate that the PLWD did not exhibit the behavior. Items were adapted from several established instruments to represent the diversity of behavioral symptoms, rather than a single conceptual construct, using approaches applied in other studies with daily measures.^33–35^ A total score was computed by summing across all endorsed items.

#### Daily psychological distress.

Daily psychological distress was measured using the Patient-Reported Outcomes Measurement Information System (PROMIS) Emotional Distress—Depression Short Form 4a and Emotional Distress—Anxiety Short Form 4a, adapted for daily diary surveys. The PROMIS Emotional Distress—Depression Short Form 4a includes four items measuring common depressive symptoms.^36,37^ Each evening, participants were asked to report, from 7:00 p.m. the previous day to 7:00 p.m. that day, the extent to which they experienced the following: “I felt worthless,” “I felt helpless,” “I felt depressed,” and “I felt hopeless.” The PROMIS Emotional Distress—Anxiety Short Form 4a includes four items assessing common anxiety symptoms. Participants were asked, for the same time frame, the extent to which they experienced the following: “I felt fearful,” “I found it hard to focus on anything other than my anxiety,” “My worries over-whelmed me,” and “I felt uneasy.” For the present analysis, a binary score was created indicating whether any of the depressive or anxious symptoms were endorsed on a given day. The binary scores were used as separate outcomes for our multilevel logistic regression models. This approach was selected to capture the occurrence of distress symptoms in daily life rather than symptom severity or clinical diagnosis. Consistent with prior research, modeling symptom occurrence allowed for the examination of within-person dynamics and transitions in distress states over time.^25^ In addition, this operationalization enhanced interpretability by allowing results to be expressed as the likelihood of experiencing a “symptom day,” which may be more informative for identifying periods of vulnerability than small fluctuations in continuous symptom scores. Changes in the likelihood of symptom days over time may reflect broader dynamic patterns (e.g., changes in the number of days with symptoms) that have been proposed as early warning signals of worsening distress.^38^

### 2.4 | Analysis

All statistical analyses were conducted using R version 4.5.1 and RStudio version 2025.09.1.0.^39^ Descriptive statistics were used to summarize demographic characteristics, the caregiving situation, financial strain, and daily psychological distress. Separate multilevel logistic regression models were estimated for daily depressive symptoms and daily anxiety symptoms to account for the nested structure of the data (daily observations nested within caregivers). Unadjusted models with a single predictor were also used to examine the associations between financial strain and caregiving-related stress with depressive and anxiety symptoms. The primary adjusted models included financial strain as the primary predictor. Age and sex were included as covariates, as these factors have previously been shown to influence caregiver stress and mental health outcomes.^40,41^ Moreover, female and younger caregivers are more likely to report high financial strain.^10,42^ The primary caregiving stressors total score was also included as a model covariate, as prior research indicates an association between caregiving costs and distress related to BPSDs.^23^ Household income was examined as a potential covariate; however, due to missing data, inclusion of this variable resulted in a reduction of the analytic sample size. Thus, household income was not included in the final models. We did not control for employment status given the dynamic relationship between employment and caregiving: Caregivers may increase paid work to cover care costs or may leave the workforce due to caregiving demands.^10^ Further, while some studies have identified differences in the experiences of financial strain according to kin relationship (i.e., spousal vs adult child),^16^ we did not include this covariate to avoid over-adjusting models due to overlap with caregiver age (i.e., adult child caregivers are, on average, younger than spousal caregivers). All predictors were included simultaneously in the primary models, and standardized effects were estimated to allow comparison of effect sizes across variables.

## 3 | RESULTS

### 3.1 | Demographics

Of the 201 participants in the sample, 186 H&L ADRD family caregivers with 3195 daily observations were included in the analyses. Twenty-four participants were excluded due to missing data on the outcomes or predictor variables. Caregivers had a mean age of 55.8 years (SD = 10.4; range: 23 to 83), and the majority were female (88.7%). More than half of family caregivers were not working (57.5%), and household income varied, with 36.0% reporting less than $25,000 annually, 43.5% reporting between $25,000 and $99,999 annually, and 8.1% reporting $100,000 or more annually. The most common kin relationship represented in the sample was caring for a biological parent (61.3%), followed by caring for a spouse (18.8%). The majority of caregivers (60.8%) did not provide care for any children, while nearly a quarter (23.7%) cared for one or two children, and a small proportion (4.4%) cared for three or more children.

PLWD had a mean age of 79.8 years (SD = 8.4; range: 59 to 98) and were predominantly female (67.7%). Most were covered by Medicare (63.4%), with an additional 19.9% covered by Medicaid. On average, PLWD had 3.35 chronic conditions (SD = 1.90), with the most common being hypertension (62.4%) and depression or anxiety (60.2%). Care recipients required assistance with 3.21 activities of daily living (SD = 2.25) and 7.39 instrumental activities of daily living (SD = 1.20). The mean caregiver-reported AD8 score was 7.1 (SD = 0.95). The most common types of behavioral symptoms reported each day were memory issues (80.4% of days), appetite issues (70.6% of days), and apathetic mood (68.0% of days). Full demographics for participants and the care recipients are reported in Tables 1 and 2. Table 3 describes BPSDs reported each day, as well as caregiver distress for each type of symptom reported.

The mean financial strain score was 9.41 (SD = 3.05, range = 3 to 15). Nearly half of caregivers (47.8%) agreed or strongly agreed that caregiving placed financial strain on them, 40.9% agreed or strongly agreed they had difficulty paying for their relative’s expenses, and 43.0% agreed or strongly agreed that they had inadequate financial resources. On average, caregivers reported experiencing depressive symptoms on over half of the 21 diary days (56%) and anxiety symptoms on nearly two-thirds of days (61.9%). Table 4 presents daily mental health descriptives for caregivers, and Table 5 reports levels of financial strain.

### 3.2 | Multilevel logistic regression models

There were statistically significant relationships between covariates and outcomes for both depressive and anxiety symptoms. To facilitate comparison across predictors, all continuous predictors were standardized prior to modeling. The reported odds ratios (ORs) and 95% confidence intervals (CIs) describe the change in odds associated with a 1 SD increase in each continuous predictor. Unadjusted models run with (1) financial strain and (2) primary caregiving stressors are presented in Tables 6 and 7. Full model results are available in Tables 8 and 9.

#### Depressive symptoms.

In the unadjusted model examining the association between financial strain and depressive symptoms, financial strain was significantly associated with daily depressive symptoms (OR = 1.73, CI: [1.07, 2.79], *p* = 0.025; marginal pseudo-*R*^2^ = 0.022, conditional pseudo-*R*^2^ = 0.755). In adjusted models, financial strain (OR = 1.75, CI: [1.07, 2.86], *p* = 0.027) remained significantly associated with daily depressive symptoms, even while including primary caregiving stressors in the model (OR = 1.62, CI: [1.44, 1.83], *p* < 0.001; marginal pseudo-*R*^2^ = 0.041, conditional pseudo-*R*^2^ = 0.771). These findings indicate that greater financial strain at baseline was associated with a 75% higher likelihood of caregivers endorsing depressive symptoms on a given day. Similarly, higher daily distress associated with primary caregiving stressors was associated with a 62% higher likelihood of endorsing depressive symptoms on the same day. Notably, ORs were largely stable between unadjusted and adjusted models when both financial strain and care stressors were included together, suggesting financial strain is distinct from other types of care stressors. Caregiver age (OR = 1.19, CI: [0.73, 1.93], *p* = 0.480) and sex (OR = 0.76, CI: [0.16, 3.55], *p* = 0.722) were not significantly associated with daily depressive symptoms. Supplementary analyses that included household income as a covariate resulted in a reduced analytic sample (*n* = 163). In this reduced sample, financial strain was not significantly associated with depressive symptoms, regardless of whether income was included. Further, in these analyses, household income was also not significantly associated with depressive symptoms.

#### Anxiety.

Similar results were observed for anxiety symptoms. In unadjusted models, financial strain was also significantly associated with daily anxiety symptoms (OR = 1.75, CI: [1.11, 2.77], *p* = 0.016; marginal pseudo-*R*^2^ = 0.026, conditional pseudo-*R*^2^ = 0.726). In adjusted models, both financial strain (OR = 1.75, CI: [1.09, 2.81], *p* = 0.020) and distress related to primary caregiving stressors (OR = 1.58, CI: [1.39, 1.79], *p* < 0.001) were significantly associated (marginal pseudo-*R*^2^ = 0.041, conditional pseudo-*R*^2^ = 0.742). Once again, as with depressive symptom models, there was very little change in ORs when models were adjusted for both types of stressors and covariates. Greater baseline financial strain was associated with a 75% higher likelihood of caregivers endorsing anxiety symptoms on a given day. At the daily level, increased caregiving stress was associated with a 58% greater likelihood of caregivers endorsing anxiety symptoms on the same day. Neither caregiver age (OR = 0.98, CI: [0.61, 1.56], *p* = 0.921) nor sex (OR = 0.96, CI: [0.22, 4.19], *p* = 0.956) was significantly associated with anxiety symptoms. Consistent with the depressive symptom model, the analytic sample was reduced when income was added, and financial strain was not significantly associated with anxiety symptoms. Household income was also not associated with anxiety in these supplementary analyses.

## 4 | DISCUSSION AND CONCLUSIONS

Findings from this analysis demonstrate that financial strain reported at baseline is significantly associated with daily depressive and anxiety symptoms among H&L ADRD family caregivers, a population that experiences disproportionately high caregiving and financial burden. Notably, financial strain demonstrated a larger standardized association with daily emotional distress than caregiving-related stress, although both were independently associated with depressive and anxiety symptoms. The absence of change in the odds of depressive and anxiety systems when models were adjusted for other care-related stressors suggests that the effect of financial strain is distinct from other types of caregiving stressors, such as BPSDs. This finding is particularly striking given the well-established link between BPSDs and caregiver depressive and anxiety symptoms and suggests that, for H&L caregivers, economic stressors may be at least as salient as caregiving-related stressors in shaping day-to-day mental health experiences.^25,43^

### 4.1 | Why financial strain is a critical issue for H&L ADRD caregivers

The findings of this study should be interpreted in the context of the disproportionate caregiving and financial burden experienced by H&L ADRD family caregivers, as well as the characteristics of the study sample. In the present sample, four out of 10 caregivers reported working for pay, compared to approximately six out of 10 caregivers nationally.^9^ This pattern may reflect the higher intensity of care provided by H&L ADRD family caregivers, as well as structural and cultural factors that shape caregiving roles. Recent estimates indicate that Latino older adults living with dementia receive 36 h of informal care on average per week, compared to 30 h among Black/African American older adults and 20 h among non-Hispanic White older adults. This equates to nearly $45,000 in care provided by Hispanic caregivers, compared to $25,000 among non-Hispanic Whites.^44^ This sample also appears to have a lower total household income than the nationally reported average among caregivers, which was $80,600 in 2023.^45^ In this sample, 35% of caregivers reported a household income below $25,000, and 44% reported a household income between $25,000 and $99,000. In the United States, the impact of more limited paid employment extends beyond income alone, since employers are the primary source of health insurance. While most care recipients in this sample were insured, this is not necessarily true of caregivers. In the United States, Latino individuals are more than twice as likely to be uninsured compared to non-Latino White individuals, which could add to financial strain for these caregivers when managing costs for their own healthcare needs alongside family caregiving costs.^46^ Limited access to employment-based healthcare, in turn, limits access to treatments for depression and anxiety despite heightened risk.

### 4.2 | Comparison of results with previous findings

Results from this study are consistent with prior studies examining financial strain and mental health, as well as what one would expect based on the Stress Process Model, with financial strain predicting daily symptoms of depression and anxiety.^18^ Whereas Sun et al. identified an association between financial strain and depressive and anxiety symptoms using a single-time-point, retrospective survey,^4^ the present study established associations between financial strain at baseline and depressive and anxiety symptoms in the subsequent 21 days. Further, unlike other studies where financial strain was found to predict depressive symptoms,^21^ the outcomes of this research reflect the daily experiences of family caregivers. While others have used micro-longitudinal approaches to examine overload and role captivity related to financial strain,^17^ we are not aware of other investigations using a daily survey method to examine associations between financial strain and depressive and anxiety symptoms.

Importantly, our findings are consistent with qualitative studies of family caregivers, which describe caregivers’ immense distress related to financial strain from caregiving. Uncertainty about future expenses and one’s own financial security, as reported in qualitative studies, may contribute to symptoms of anxiety.^7,8^ This uncertainty may be exacerbated among H&L caregivers, who may provide care for a greater length of time due to longer lifespans among US H&L populations.^47^ Feelings of hopelessness related to the inevitability of costs may explain links to depressive symptomology,^8^ as well as the conflict between wanting to provide excellent care that may come at a financial cost, even when material resources are limited.^11^ These experiences may be exacerbated among younger caregivers as they navigate early-career roles, as well as the impact of caregiving on long-term employability and savings.^48^

### 4.3 | Limitations

This study had several limitations. First, financial strain was measured at baseline, rather than day to day. The extent to which financial strain may fluctuate among caregivers is unknown, as is whether this covaries with depressive and anxiety symptoms. Further, the analysis did not examine potential interactions between covariates, such as how differences in caregiver age may affect the relationship between financial strain and daily mental health experiences, given potential differences in caregiving experiences across caregiver life stages. Rather, this study provides initial evidence that financial strain is a key, potentially modifiable risk factor for caregiver depression and anxiety. Similarly, this study did not examine differences based on whether the caregiver was born in the United States or outside the United States, nor other aspects of acculturation, though these factors are known to affect mental health.^49^ While the results from the present study suggest financial strain explains variance in daily depressive and anxiety symptoms, future research should consider long-term impacts on caregiver mental health over months- and years-long periods. Finally, household income was not included in the primary models due to missing data and the resulting reduction in sample size. Future research with larger samples should examine the joint and independent contributions of objective measures and other proxies of socioeconomic status to H&L caregivers’ mental health. Moreover, we acknowledge that there are more nuanced measures of objective financial strain than household income. For example, our measure used wide income bands (e.g., $25,000 to$99,000), which may conceal differences among caregivers. Future research should account for differences in household size and region and examine narrower income bands to better capture the impact of objective financial strain on caregiver mental health.

### 4.4 | Implications and future research

This study found that financial strain affected the daily mental health experiences of family caregivers beyond that of more studied predictors, such as distress related to BPSDs. This suggests that the overall exclusion of financial strain from studies to understand factors that increase or mitigate the risk of depression and anxiety in ADRD family caregivers overlooks a potentially critical driver of these outcomes. This is consistent with listening sessions conducted with family caregivers to inform the US 2022 National Strategy on Caregiving, in which caregivers emphasized the impact of financial strain on their experiences.^50^ Future research should examine longitudinal changes in financial strain and emotional distress and evaluate the potential for interventions aimed at reducing financial strain to improve caregiver well-being in this high-risk population. Clinical implications of our findings include the need to screen family caregivers for financial strain so they can be connected to services to ensure access to affordable mental health treatment and support financial security. This includes referral to financial education programs to help caregivers plan, such as preparing for the likelihood that a family member’s condition will span many years and that resource demands are likely to change over time. Interventions to support caregivers in managing the challenges of financial strain, including its emotional toll, are emerging.^51^ As these programs develop, interventions must recognize that caregivers lead integrated lives, where sources of financial strain are multicomponent and reflect complex costs related to the disease as well as difficult choices about how to manage employment and household resources. While objective financial strain is distinct from psychological financial strain, objective sources of strain also serve as sources of worry. As such, we also recommend policy-level interventions, such as more flexible workplace policies, state- or federal-level paid family leave policies, and more robust tax credits to reduce the impact of out-of-pocket costs of care.

## 5 | CONCLUSION

Financial strain appears to play an important role in the mental health of family caregivers of PLWD, with especially meaningful implications for H&L caregivers who face disproportionate prevalence of ADRD and caregiving burden. In this study, caregivers reporting higher subjective financial strain were more likely to experience depressive and anxiety symptoms on a given day. Further, financial strain may have a stronger influence on daily mental health experiences than distress related to BPSDs among H&L ADRD caregivers. Future work should examine how financial strain changes over time and identify culturally responsive approaches to reducing both financial hardship and its emotional toll among H&L caregivers.

## Supplementary Material

ICJME author disclosure

Additional supporting information can be found online in the Supporting Information section at the end of this article.

## Figures and Tables

**TABLE 1:** Hispanic and Latino ADRD family caregiver demographics (*N* = 186).

Variable	Mean ± SD (range)	*N*(%)
Age (years)	55.8 ± 10.4 (23 to 83)	

Sex		
Female		165 (88.7)
Male		21 (11.3)

Employment status		
Not working		107 (57.5)
Working		74 (39.8)
Missing		5 (2.7)

Household income		
High ($100,000+)		15 (8.1)
Middle ($25,000 to $99,000)		81 (43.5)
Low (less than $25,000)		67 (36.0)
Missing		23 (12.4)

Provide care to others		
No		146 (78.5)
Yes		37 (19.9)
Missing		3 (1.6)

Number of children providing care for		
0		113 (60.8)
1 or 2		44 (23.7)
3 or more		8 (4.4)

Relationship to care recipient		
Biological grandparent		9 (4.8%)
Biological parent		114 (61.3%)
Husband/wife/spouse		35 (18.8%)
Other (specify)		15 (8.1%)
Parent-in-law		10 (5.4%)
Stepparent		3 (1.6%)

Abbreviation: SD, standard deviation.

**TABLE 2:** Hispanic and Latino ADRD care recipient demographics (*N* = 186).

Variable	Mean ± SD (range)	*N* (%)
Age (years)	79.8 ± 8.43 (59 to 98)	

Sex		
Female		126 (67.7)
Male		53 (28.5)

AD 8 score (frequency)		
3		1 (0.5)
4		2 (1.1)
5		10 (5.4)
6		20 (10.8)
7		77 (41.4)
8		76 (40.9)

AD8 score	7.14 ± 0.95	

Insurance		
Employer insurance		5 (2.7)
Medicaid		37 (19.9)
Medicare		118 (63.4)
Veteran’s insurance		3 (1.6)
No insurance		8 (4.3)
Not sure		2 (1.1)
Other (specify)		11 (15.9)
Missing		2 (1.1)

Chronic conditions		
Hypertension/HBP		116 (62.4%)
Atrial fibrillation (A-fib)		18 (9.7%)
Diabetes		67 (36.0%)
Previous stroke/TIA		25 (13.4%)
Heart failure		30 (16.1%)
Joint problems		96 (51.6%)
Kidney problems		23 (12.4%)
Lung disease/COPD		13 (7.0%)
Cancer or malignancy^a^		14 (7.5%)
Depression or anxiety		112 (60.2%)
Substance abuse		5 (2.7%)
Schizophrenia^b^		6 (3.2%)
Hearing loss or deafness		55 (29.6%)
Severe vision problems		31 (16.7%)
Parkinson’s/ALS^c^		13 (7.0%)

Number of chronic conditions	3.35 ± 1.90 (0 to 9)	

Number of ADLs	3.21 ± 2.25 (0 to 6)	

Number of IADLs	7.39 ± 1.20 (1 to 8)	

Abbreviations: AD8, The AD8: The Washington University Dementia Screening Test; ADL, activities of daily living; ALS, amyotrophic lateral sclerosis; COPD, chronic obstructive pulmonary disease; HBP, high blood pressure; IADL, instrumental activities of daily living; SD, standard deviation; TIA, transient ischemic attack.

a Non-skin cancer or malignancy.

b Or other serious mental illness.

c Or other neuromuscular condition.

**TABLE 3:** Hispanic and Latino ADRD care recipient daily behavioral and psychological symptoms of dementia (*N* = 186).

BPSD	Daily observations of behavior occurring Yes (%)	Caregiver distress Mean ± SD
Restlessness	1703 (53.3%)	1.45 ± 1.42
Memory	2570 (80.4%)	1.66 ± 1.37
Mood depressed	1480 (46.3%)	1.22 ± 1.28
Mood anxiety	1710 (53.5%)	1.44 ± 1.38
Mood euphoria	1172 (36.7%)	0.73 ± 1.05
Mood apathy	2174 (68.0%)	1.37 ± 1.37
Care-resistant behaviors	2045 (64.0%)	1.82 ± 1.42
Property destruction	734 (23.0%)	1.25 ± 1.47
Hallucinations	1511 (47.3%)	1.51 ± 1.46
Delusions	1511 (47.3%)	1.59 ± 1.41
Verbal aggression	945 (29.6%)	1.44 ± 1.43
Physical aggression	703 (22.0%)	1.20 ± 1.52
Disinhibition	650 (20.3%)	0.98 ± 1.23
Wandering	1745 (54.6%)	1.48 ± 1.47
Repetitive verbal behaviors	1237 (38.7%)	1.27 ± 1.37
Repetitive motor behaviors	2057 (64.4%)	1.61 ± 1.41
Irritability	1745 (54.6%)	1.47 ± 1.34
Impulsive behaviors	984 (30.8%)	1.23 ± 1.32
Appetite	2256 (70.6%)	1.51 ± 1.37
Other	1204 (37.7%)	1.42 ± 1.54

*Note*: BPSD appraisal scale range 0 to 5 (0 = not bothersome; 5 = extremely bothersome).

Abbreviations: BPSD, behavioral and psychological symptoms of dementia; SD, standard deviation.

**TABLE 4:** Daily descriptives for Hispanic and Latino ADRD caregivers (*n* = 3195).

Variable	Mean ± SD	*n* (%)
Depressive symptoms reported		
No		1407 (44.0)
Yes		1788 (56.0)

Anxiety symptoms reported		
No		1218 (38.1)
Yes		1977 (61.9)

BPSD stress	13.6 ± 14.0 (0 to 96)	

Abbreviations: BPSD, behavioral and psychological symptoms of dementia; SD, standard deviation.

**TABLE 5:** Financial strain descriptives for Hispanic and Latino ADRD caregivers (*N* = 186).

Variable	Mean ± SD	*n* (%)
Financial strain score	9.41 ± 3.05 (3 to 15)	

Financial resources are adequate		
Strongly disagree		39 (21.0)
Disagree		41 (22.0)
Neither agree/disagree		41 (22.0)
Agree		43 (23.1)
Strongly agree		22 (11.8)

It is difficult to pay for my relative		
Strongly disagree		30 (16.1)
Disagree		36 (19.4)
Neither agree/disagree		43 (23.1)
Agree		45 (24.2)
Strongly agree		31 (16.7)
Missing		1 (0.5)

Care for my relative puts financial strain on me		
Strongly disagree		30 (16.1)
Disagree		27 (14.5)
Neither agree/disagree		38 (20.4)
Agree		49 (26.3)
Strongly agree		40 (21.5)
Missing		2 (1.1)

Abbreviation: SD, standard deviation.

**TABLE 6:** Unadjusted predictors in depressive symptoms multilevel binary logistic model (*n* = 3195).

Predictors	Odds ratio	95% CI	*p* value
Intercept	1.75	[1.09, 2.82]	0.021*

Financial strain	1.73	[1.07, 2.79]	0.025*

Random effects			
Random intercept variance	3.29		
ICC	0.75		

Variance explained			
Marginal *R*^2^	0.022		
Conditional *R*^2^	0.755		

Intercept	1.80	[1.09, 2.96]	0.021*

BPSD stress	1.62	[1.44, 1.84]	<0.001***

Random effects			
Random intercept variance	3.29		
ICC	0.77		

Variance explained			
Marginal *R*^2^	0.016		
Conditional *R*^2^	0.770		

Abbreviations: BPSD, behavioral and psychological symptoms of dementia; CI, confidence interval; ICC, Intraclass correlation coefficient.

* *p* < 0.05

** *p* < 0.01,

*** *p* < 0.001.

**TABLE 7:** Unadjusted predictors in anxiety multilevel binary logistic model (*n* = 3195).

Predictors	Odds ratio	95% CI	*p* value
Intercept	3.16	[1.98, 5.05]	<0.001***

Financial strain	1.75	[1.11, 2.77]	0.016*

Random effects			
Random intercept variance	3.29		
ICC	0.72		

Variance explained			
Marginal *R*^2^	0.026		
Conditional *R*^2^	0.726		

Intercept	3.24	[2.04, 5.14]	<0.001***

BPSD stress	1.58	[1.40, 1.79]	<0.001***

Random effects			
Random intercept variance	3.29		
ICC	0.73		

Variance explained			
Marginal *R*^2^	0.017		
Conditional *R*^2^	0.737		

Abbreviations: BPSD, behavioral and psychological symptoms of dementia; CI, confidence interval; ICC, intraclass correlation coefficient.

* *p* < 0.05,

** *p* < 0.01,

*** *p* < 0.001.

**TABLE 8:** Depressive symptoms multilevel binary logistic model (*n* = 3195).

Predictors	Odds ratio	95% CI	*p* value
Intercept	1.88	[1.11, 3.17]	0.018*

Caregiver age	1.19	[0.73, 1.93]	0.480

Caregiver gender (male)	0.76	[0.16, 3.55]	0.722

Financial strain	1.75	[1.07, 2.86]	0.027*

BPSD stress	1.62	[1.44, 1.83]	<0.001***

Random effects			
Random intercept variance	10.50		
ICC	0.76		

Variance explained			
Marginal *R*^2^	0.041		
Conditional *R*^2^	0.771		

*Note*: Marginal and conditional *R*^2^ values represent pseudo-*R*^2^ measures of explained variance for mixed models.

Abbreviations: BPSD, behavioral and psychological symptoms of dementia; CI, confidence interval; ICC, intraclass correlation coefficient.

* *p* < 0.05,

** *p* < 0.01,

*** *p* < 0.001.

**TABLE 9:** Anxiety multilevel binary logistic model (*n* = 3195).

Predictor	Odds ratio	95% CI	*p* value
Intercept	3.34	[2.00, 5.58]	<0.001***

Caregiver age	0.98	[0.61, 1.56]	0.921

Caregiver gender (male)	0.96	[0.22, 4.19]	0.956

Financial strain	1.75	[1.09, 2.81]	0.020*

BPSD stress	1.58	[1.39, 1.79]	<0.001***

Random effects			
Random intercept variance	8.96		
ICC	0.73		

Variance explained			
Marginal *R*^2^	0.041		
Conditional *R*^2^	0.742		

*Note*: Marginal and conditional *R*^2^ values represent pseudo-*R*^2^ measures of explained variance for mixed models.

Abbreviations: BPSD, behavioral and psychological symptoms of dementia; CI, confidence interval; ICC, intraclass correlation coefficient.

* *p* < 0.05,

** *p* < 0.01,

*** *p* < 0.001.

## Data Availability

Data from this study will be publicly deposited in the National Archive of Computerized Data on Aging at the end of the parent study.

## Highlights

- Higher levels of subjective financial strain predicted significantly greater odds of daily depressive and anxiety symptoms among Hispanic and Latino dementia caregivers.
- Financial strain demonstrated associations with daily emotional distress that were comparable to daily behavioral and psychological symptoms of dementia.
- Findings underscore the need for interventions that directly address financial hardship to improve caregivers’ day-to-day mental health.

## RESEARCH IN CONTEXT

1. **Systematic review:** The authors reviewed the literature on financial strain and mental health among family caregivers of PLWD using traditional sources (e.g., PubMed, PsycINFO). While financial strain in dementia caregiving has been examined, prior studies primarily used traditional (i.e., one-time survey) cross-sectional or retrospective designs, and relatively few examined financial strain and daily mental health experiences among Hispanic and Latino dementia caregivers. Relevant studies are cited appropriately.
2. **Interpretation:** Our findings suggest that subjective financial strain is significantly associated with daily psychological distress among Hispanic and Latino dementia caregivers. The size of associations is comparable to known stressors, such as behavioral and psychological symptoms of dementia.
3. **Future directions:** Future research should examine how financial strain fluctuates over time, identify mechanisms linking financial strain to daily emotional distress, and evaluate culturally responsive interventions to enhance the financial and psychological well-being of Hispanic and Latino dementia caregivers.
